# UBA1 drives cell cycle to promote malignancy and serves as a therapeutic target in Ewing Sarcoma

**DOI:** 10.1186/s12885-026-16272-z

**Published:** 2026-06-06

**Authors:** Shangjiang Yu, Runyi Jiang, Lei Zhou, Anshun Wu, Haifeng Wei, Shaohui He, Jianru Xiao

**Affiliations:** 1 School of Health Science and Engineering, University of Shanghai for Science and Technology, Shanghai, 200093 China; 2 Spinal Tumor Center, Department of Orthopaedic Oncology, Changzheng Hospital, Naval Medical University (Second Military Medical University), Shanghai, 200003 China; 3 Clinical Medical College, North China University of Science and Technology, Tangshan, 063210 China

**Keywords:** UBA1, Ewing Sarcoma, Cell cycle

## Abstract

**Background:**

Ewing sarcoma (ES) is an aggressive malignancy with high metastatic potential and poor prognosis, necessitating novel biomarkers and therapeutic targets. This study investigates the role of the ubiquitin-activating enzyme E1 (UBA1) in ES pathogenesis and its clinical relevance.

**Methods:**

Bioinformatic analysis of GEO datasets was performed. UBA1 expression was validated in clinical samples and ES cell lines (A673, RDES) via Western blot and qPCR. To investigate the functional role and therapeutic potential of UBA1 in ES, we performed siRNA-mediated knockdown followed by a series of in vitro functional assays, including assessments of proliferation, colony formation, migration, invasion, cell cycle distribution, and EdU incorporation. Mechanistic studies were subsequently conducted through the WB experiment. In vivo experiments were carried out using a xenograft mouse model to evaluate the effect of UBA1 depletion on tumor growth. Additionally, the UBA1-specific inhibitor TAK-243 was used to assess the therapeutic potential of targeting UBA1.

**Results:**

UBA1 expression was significantly elevated in ES tissues, and high UBA1 levels correlated with poor prognosis. Knockdown of UBA1 suppressed malignant phenotypes, including proliferation, colony formation, migration, and invasion, while inducing cell cycle arrest and inhibiting DNA synthesis. Mechanistically, UBA1 depletion led to dysregulation of the G2/M checkpoint pathway, consistent with Gene Set Enrichment Analysis (GSEA) predictions. In vivo experiments confirmed the oncogenic role of UBA1, and treatment with the UBA1 inhibitor TAK-243 recapitulated the effects observed upon UBA1 knockdown.

**Conclusion:**

Targeting UBA1 disrupts cell cycle progression and inhibits proliferation in ES, underscoring its therapeutic potential.

## Introduction

Among primary malignancies of bone, ES ranks second in frequency and shows a marked predilection for children and adolescents [1–3]. Clinically, its aggressive nature is reflected in the high rate of distant metastases at presentation—affecting up to 80% of patients—which contributes to a 5-year survival rate below 30% for those with advanced disease [4–6]. Although ES is molecularly defined by pathognomonic EWS-FLI1 or EWS-ERG fusions [7, 8], therapeutic efforts to directly target these oncoproteins have met with limited success. Compounding this challenge, the profoundly immunosuppressive tumor microenvironment further undermines the effectiveness of immunotherapeutic approaches [9, 10]. Therefore, elucidating the molecular mechanisms underlying ES progression and identifying novel prognostic markers and therapeutic targets remain critical and urgent research priorities.

Recent studies have implicated the ubiquitin-proteasome system (UPS) in regulating the malignant behaviors of ES. Evidence supporting the UPS as a therapeutic target in ES comes from studies demonstrating that MDM2-mediated p53 degradation promotes chemoresistance, and that USP9X-dependent stabilization of EWS-FLI1 enhances tumor cell survival [11, 12]. As the master initiator of the ubiquitination cascade, UBA1 acts as the “gatekeeper” of the entire pathway. Given that UBA1 governs fundamental oncogenic processes including cell cycle regulation and DNA damage repair across various cancers [13, 14], it is therefore a compelling therapeutic target. This notion is supported by the potent anti-tumor activity of the specific UBA1 inhibitor TAK-243, particularly against hematological malignancies [15–17]. However, the expression pattern, clinical relevance, and specific functional roles of UBA1 in ES remain entirely unexplored.

To address this knowledge gap, we systematically characterized UBA1 expression and prognostic value in ES clinical specimens and cell lines, and further delineated its functional role in ES malignant phenotypes while probing the underlying mechanisms. These research results indicate that UBA1 plays a significant role in the malignant biology of ES. It not only can predict the prognosis of patients, but also may become a therapeutic target.

## Materials and methods

### Public data analysis

Public databases, including the GEO and the International Cancer Genome Consortium (ICGC), provide mRNA sequencing data from ES patients with clinical information for analysis. This study utilized the GSE17674 dataset from the GEO database to analyze the differential expression of UBA1 between ES tumor tissues and normal control tissues. Additionally, the GSE63155 and GSE63156 datasets from GEO, alongside ES next-generation sequencing data from the ICGC database, were combined to analyze the correlation between UBA1 expression and patient prognosis. All datasets were normalized prior to analysis. Based on UBA1 expression levels, ES patients within each dataset were stratified into high and low UBA1 expression groups. Differentially Expressed Genes (DEGs) between these two groups were identified. GSEA was subsequently performed to investigate the potential biological processes involving UBA1 in the pathogenesis and progression of ES.

### Tissue samples and cell lines

A total of 12 ES tissue specimens (including tumor tissues and adjacent non-cancerous tissues) used in this study were obtained from Shanghai Changzheng Hospital, collected between May and October 2024, with the approval of the hospital’s ethics committee. The study was conducted in accordance with the Declaration of Helsinki and approved by the Institutional Review Board of Changzheng Hospital. Human BMSC and human ES cell lines (A673, RDES) were procured from Shanghai Fuheng Biology Co., Ltd., and authenticated by short tandem repeat (STR) profiling. BMSC were cultured in α-MEM medium (Gibco, USA) supplemented with 10% fetal bovine serum (Gibco, USA) and passaging performed weekly. A673 cells were cultured in DMEM medium (Gibco, USA) containing 10% FBS and passaged every 2–3 days. RDES cells were cultured in RPMI 1640 medium (Gibco, USA) supplemented with 10% FBS and passaged every 2–3 days. All cells were maintained in an incubator at 37 °C with 5% CO₂.

### Cell transfection

Log-phase cells in good growth condition were seeded into 12-well plates. When cell density reached approximately 70%, 100 µl of opti-MEM (Gibco, USA) was aliquoted into an RNase-free EP tube. The siRNA was diluted according to the manufacturer’s instructions, and 2 µl was added to the EP tube, followed by 6 µl of transfection reagent. The mixture was vortexed thoroughly and incubated at room temperature for 15 min. After 15 min, the medium in the plate was replaced with serum-free medium, and the transfection complex mixture was added dropwise. After 4–6 h, the medium was replaced with complete medium. Cells were harvested 48 h post-transfection for subsequent analysis. The RNA oligonucleotide sequences for UBA1 were: siUBA1-1: 5′-GGGAUGUCACGAAGUUAAAGU-3′; siUBA1-2: 5′-GUGCUAUGGUUUCUAUGGUUA-3′; siUBA1-3: 5′-CCACUGCCUUCUACCUUGU-3′. The sequence for the negative control RNA oligonucleotide was: 5′-UUCUCCGAACGUGUCACGU-3′.

### Quantitative Real‑Time (qRT)‑Polymerase Chain Reaction (PCR)

Total RNA was isolated from cell lines using RNA extraction kit (Accurate Biology, China), and its concentration was measured. The extracted RNA was reverse-transcribed into cDNA using reverse transcription kit (Accurate Biology, China). Finally, qRT-PCR was performed using specific primers and ChamQ Universal SYBR qPCR Master Mix (Vazyme, China). Upon completion of the program, data were exported, and the differential expression of UBA1 was analyzed using the 2 − ΔΔCT method. Primer sequences were as follows: UBA1-F: 5′-TCCTCACAGAGGACAAGTGCCT-3′, UBA1-R: 5′-CTTGAGCAGCTCACAGCCAATG-3′; Actin-F: 5′-GGCTGTATTCCCCTCCATCG-3′, Actin-R: 5′-CCAGTTGGTAACAATGCCATGT-3′. The qPCR reaction conditions were: 95 °C for 5 min; followed by 40 cycles of 95 °C for 30 s, 55 °C for 30 s, and 72 °C for 30 s.

### Western Blot (WB) and antibodies

Cells from each group were collected, and total protein was extracted using RIPA lysis buffer (Epizyme Biotech, China) containing protease inhibitors (MCE, USA). Protein concentration was quantified using the BCA method (Thermo, USA). Equal amounts of protein (20 µg per group) were loaded onto a PAGE gel (Vazyme, China) for electrophoresis separation, followed by transfer onto a nitrocellulose membrane. The membrane was blocked with 5% skim milk at room temperature for 60 min and subsequently incubated overnight at 4 °C with a primary antibody solution prepared in 5% (w/v) BSA. The following day, the membrane was incubated with an HRP-conjugated anti-rabbit secondary antibody at room temperature for 2 h. Chemiluminescent detection was performed using a ChemiDoc XRS+ imaging system, and the resulting images were analyzed using Image Lab software. Antibodies were sourced from these suppliers, including Abcam (anti-UBA1), CST (anti-CDK1, anti-p-CDK1 Tyr15, anti- Cyclin B1) and Abmart (anti-GAPDH, anti-β-actin, HRP-conjugated anti-rabbit, HRP-conjugated anti-mouse).

### Cell growth and proliferation assays

The transfected A673 and RDES cell lines were seeded in 96-well plates at a density of 1 × 10³ cells per well. Cell proliferation was assessed using the CCK-8 assay at days 0, 1, 3, 5, and 7. At each time point, the culture medium was replaced with fresh DMEM containing 10% (v/v) CCK-8 reagent (Biosharp, China), followed by incubation for 1 h at 37 °C. Absorbance was then measured at 450 nm using a microplate reader (bio Tek, USA).

For colony formation assays, transfected cells were plated in 6-well plates at a density of 1 × 10³ cells per well and cultured for 10 days. The resulting colonies were fixed with 4% paraformaldehyde for 30 min, stained with 0.1% crystal violet for 20 min, and imaged.

### EdU cell proliferation assay

Transfected A673 and RDES cells were seeded into 12-well plates at a density of 1 × 10⁵ cells per well and cultured for 24 h. EdU labeling was then performed using an EdU assay kit (Beyotime, China) following the manufacturer’s protocol. Briefly, cells were incubated with EdU for 2 h, fixed with 4% paraformaldehyde, permeabilized, and then reacted with the click reaction mixture to visualize DNA synthesis, followed by counterstaining of nuclei with Hoechst 33,342 under dark conditions. Fluorescence images were acquired using a ZEISS Axioscope 5 microscope system, and data were analyzed with ZEISS ZEN 3.13 software.

### Flow cytometry

Transfected A673 and RDES cells were seeded into 12-well plates at a density of 1 × 10⁵ cells per well and cultured until reaching approximately 70% confluence. Cells were then harvested by trypsinization, washed twice with ice-cold PBS. After fixation, cells were stained with propidium iodide (PI) staining solution (Beyotime, China) according to the manufacturer’s protocol. Briefly, cells were incubated with RNase A and PI for 30 min at 37 °C in the dark. DNA content was measured using a CyAn ADP flow cytometer (Beckman Coulter, USA). Cell cycle distribution was analyzed using ModFit LT software (Verity Software House). All experiments were performed in triplicate, and representative histograms were shown.

### Cell migration and invasion assays

Cells were seeded in the upper chamber of Transwell plates at a density of 2 × 10⁵ cells per well in serum-free medium (DMEM or RPMI 1640, as appropriate). The lower chamber was filled with medium containing 20% FBS as a chemoattractant. For the migration assay, uncoated Transwell inserts (Corning, USA) were used, whereas for the invasion assay, inserts were pre-coated with Matrigel to simulate the extracellular matrix. Following a 48-hour incubation, cells that migrated or invaded to the lower surface were fixed with 4% paraformaldehyde and stained with 0.1% crystal violet for 20 min for visualization and quantification.

### Construction and production of lentivirus

shRNA sequences targeting UBA1 were designed based on the previously validated siRNA target sequences. Each shRNA was cloned into the pLKO.1 vector as a hairpin structure consisting of the sense and antisense strands separated by a loop. Lentiviruses were generated by co-transfecting the lentiviral vector and two packaging vectors (psPAX2 and pMD2.G) into 293T cells at a ratio of 3:2:1 using PEI transfection reagent (Polyscience, USA). The supernatant was collected 48 h post-transfection and concentrated by ultracentrifugation.

### Construction of stable cell lines

To establish UBA1-silencing stable cell lines, A673 and RDES cells were transduced with shUBA1 or control lentivirus for 48 h, followed by selection with 1 µg/mL puromycin for 5 days. The surviving cells were expanded and maintained as stable polyclonal cell lines.

### In vivo studies

Female athymic BALB/c nude mice (5 weeks old) were purchased from Shanghai Jihui Laboratory Animal Care Co., Ltd., China. All animal protocols were approved by the Institutional Review Board of Changzheng Hospital (Naval Medical University). Two independent xenograft experiments were performed to evaluate the effects of UBA1 knockdown and TAK-243 (MCE, USA) treatment on ES tumor growth.

For the UBA1 knockdown experiment, A673 cells (1 × 10⁶) stably expressing shUBA1 or shNC were injected subcutaneously into the right flank of nude mice. Tumor growth was monitored every three days using a caliper, and tumor volume was calculated as (length × width²) / 2. At the end of the experiment (2 weeks post‑injection), all mice were deeply anesthetized by intraperitoneal injection of pentobarbital sodium (50 mg/kg; Sinopharm, China). The depth of anesthesia was confirmed by the absence of a pedal withdrawal reflex (toe pinch). Once fully unconscious, mice were euthanized by cervical dislocation. Tumors were excised, weighed, and photographed.

For the TAK‑243 treatment experiment, A673 cells (1 × 10⁶) were injected subcutaneously into the right flank of nude mice. When tumors became palpable, mice were randomly divided into two groups: DMSO control and TAK‑243 treatment. TAK‑243 was administered via intraperitoneal injection at 10 mg/kg once every three days for a total of three treatments. Tumor volumes were measured concurrently. Three days after the final dose, mice were euthanized using the same method as described above for the knockdown experiment, and tumors were excised, weighed, and photographed.

### Statistical analysis

Statistical analyses were performed using GraphPad Prism 10.0. Survival analysis was conducted by plotting Kaplan-Meier (KM) curves and using the log-rank test. Data are presented as the mean ± SD from at least three independent experiments. Statistical significance between two groups was assessed using Student’s t-test, while comparisons among multiple groups were analyzed by one-way ANOVA. A two-sided P-value of less than 0.05 was considered statistically significant (*P < 0.05; **P < 0.01; ***P < 0.001).

## Results

### UBA1 is highly expressed in ES and associated with poor prognosis

**Fig. 1:**
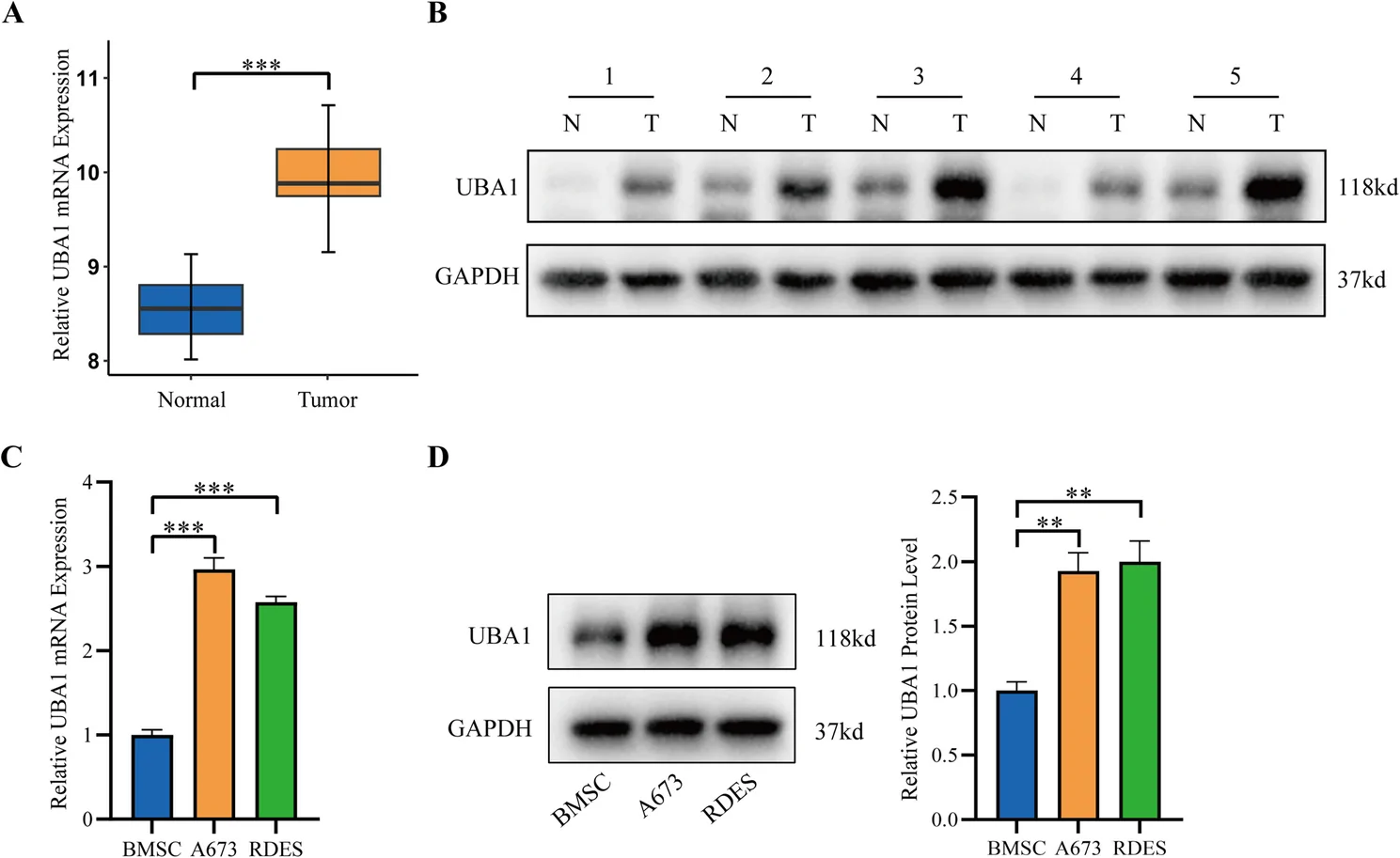
Expression levels of UBA1 in Ewing Sarcoma. **A** mRNA expression level of UBA1 in Ewing sarcoma and normal tissues from the GSE17674 sequencing dataset. **B** Representative Western blot images showing UBA1 protein expression in five paired ES and normal tissues. **C** mRNA expression level of UBA1 in Ewing sarcoma cell lines (A673, RDES) and BMSC. **D** Protein expression level of UBA1 in Ewing sarcoma cell lines (A673, RDES) and BMSC

Analysis of the GSE17674 dataset revealed that the mRNA expression level of UBA1 was significantly upregulated in ES tumor tissues compared to normal controls (*P* < 0.001, Fig. 1A). This finding was substantiated at the protein level through Western blot analysis of five paired clinical samples, which demonstrated consistently higher UBA1 expression in tumor tissues compared to corresponding normal tissues. (Fig. 1B). Furthermore, our experimental results demonstrated that UBA1 expression was significantly higher at both the mRNA and protein levels in ES cell lines than in the control BMSC cells (*P* < 0.01, Fig. 1C and D). These findings suggest that UBA1 is highly expressed in ES.

**Fig. 2:**
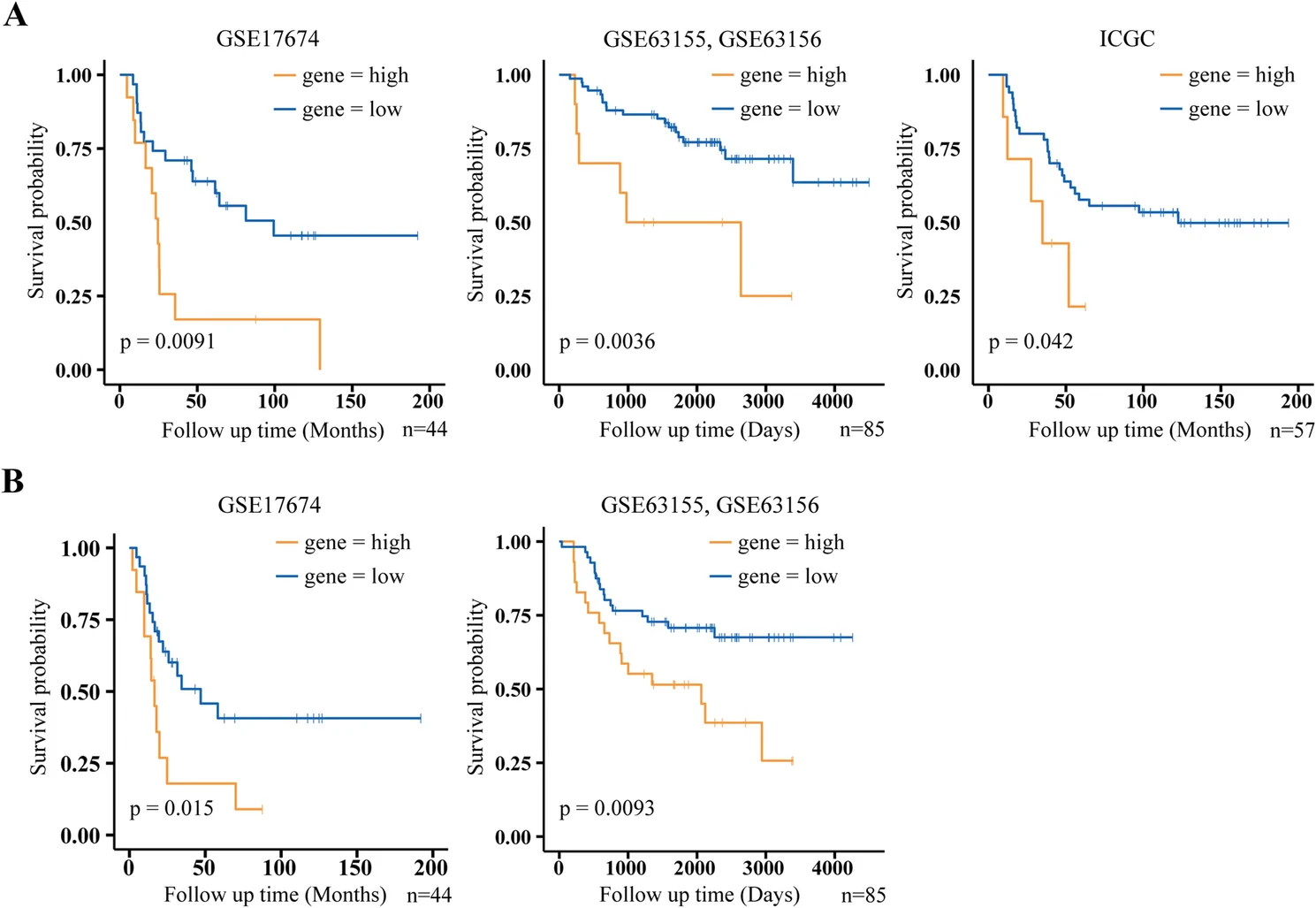
Comparison of OS and EFS between patients with high and low UBA1 expression. **A** Overall survival (OS) analysis of patients with high versus low UBA1 expression in the GSE17674, combined GSE63155 and GSE63156 datasets, and the ICGC database. **B** Event-free survival (EFS) analysis of patients with high versus low UBA1 expression in the GSE17674, combined GSE63155 and GSE63156 datasets

To investigate the impact of UBA1 expression levels on the prognosis of ES patients, we performed a pooled analysis of the GSE17674, GSE63155, and GSE63156 datasets, combined with data from the ICGC database, to assess the association between UBA1 expression and patient outcomes. The analysis revealed that patients with high UBA1 expression had a significantly shorter overall survival (OS) compared to those with low UBA1 expression (*P* < 0.05, Fig. 2A). Similarly, in the event-free survival (EFS) analysis, high UBA1 expression was also associated with a poorer clinical outcome (*P* < 0.05, Fig. 2B).

### Enrichment analysis of UBA1-associated genes in ES

**Fig. 3:**
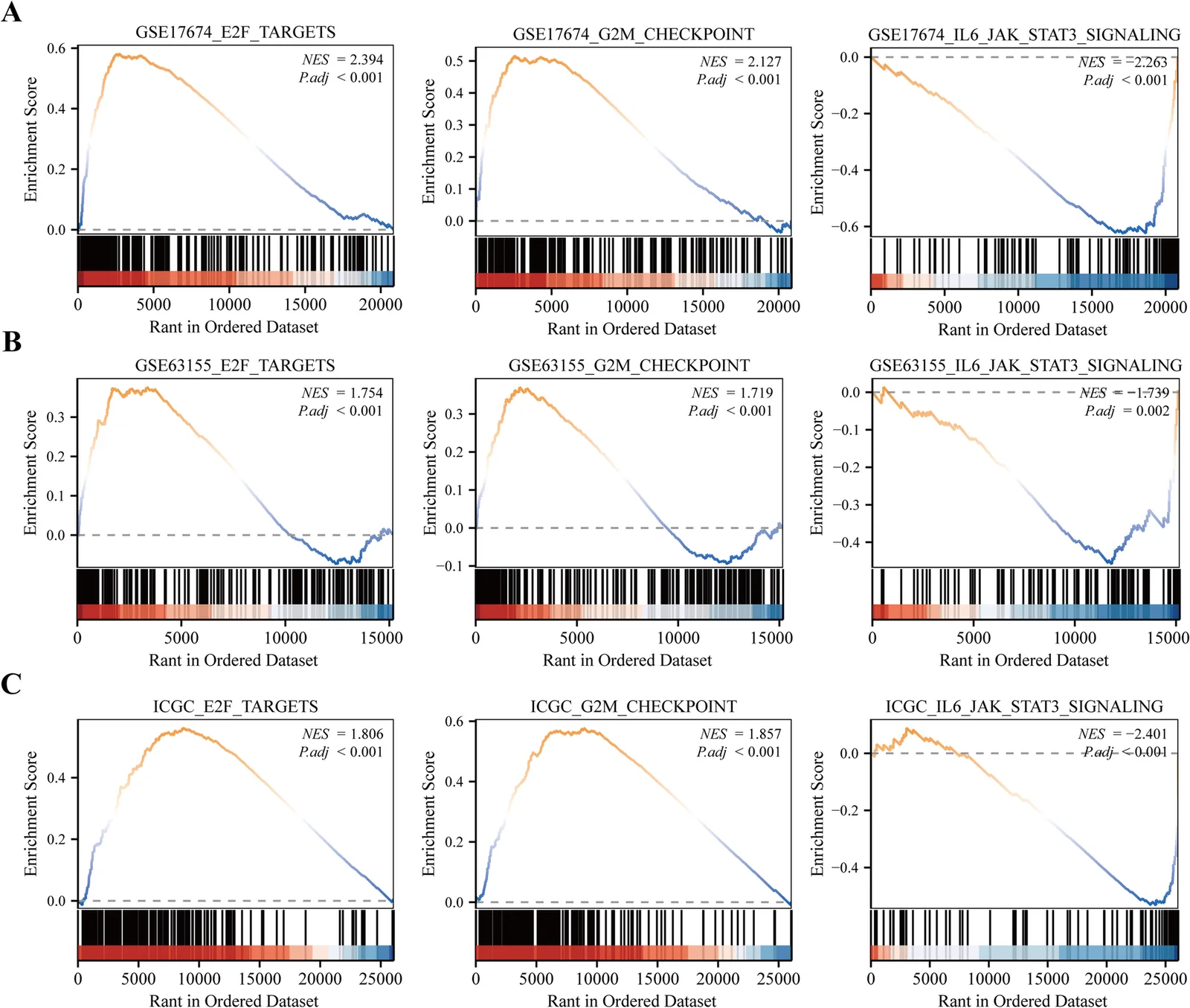
Enrichment analysis of UBA1-associated genes in Ewing Sarcoma. Gene Set Enrichment Analysis (GSEA) of the (**A**) GSE17674, (**B**) GSE63155, and (**C**) ICGC datasets

To further investigate the multifaceted roles of UBA1 in ES pathogenesis, we performed GSEA, which identified several key signaling pathways associated with UBA1 (Fig. 3A-C).

These included the E2F pathway, the G2/M DNA damage checkpoint and the IL-6-JAK-STAT3 signaling pathway. These findings suggest that high UBA1 expression drives ES progression and poor prognosis through coordinated regulation of cell cycle progression and survival signaling by promoting the G1/S phase transition via the E2F pathway, facilitating mitotic progression through the G2/M checkpoint, and mediating immune evasion and drug resistance via the IL-6-JAK-STAT3 signaling axis. These bioinformatic predictions prompted us to experimentally validate whether UBA1 indeed regulates these core oncogenic processes in ES cells.

### UBA1 knockdown impairs cell cycle progression and proliferation in ES cells

**Fig. 4:**
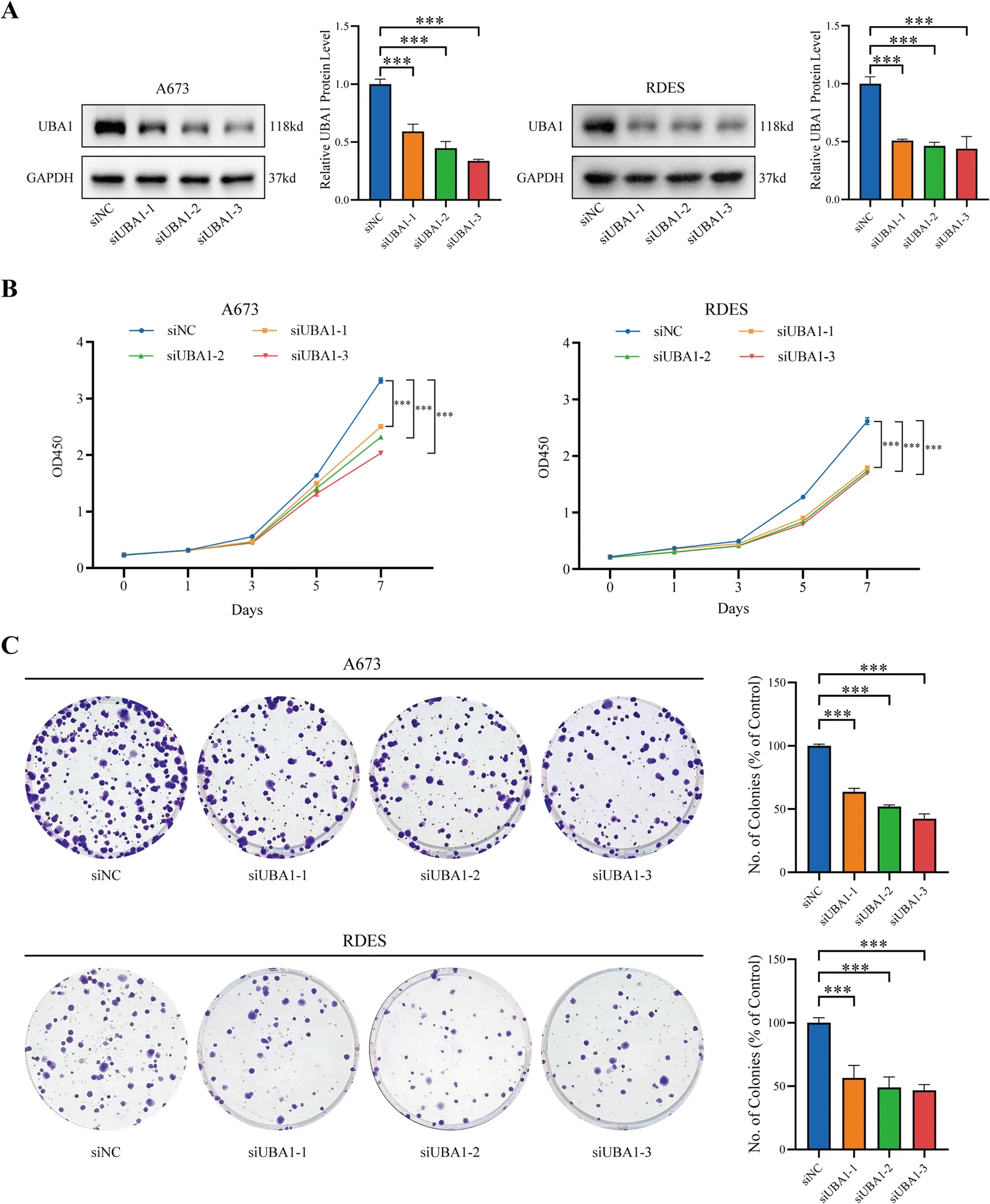
Effect of UBA1 knockdown on the proliferative capacity of ES cell lines. **A** Western blot analysis of UBA1 protein knockdown efficiency in A673 and RDES cells. **B** CCK-8 assay assessing the effect of UBA1 knockdown on the proliferation of A673 and RDES cells. **C** Colony formation assay evaluating the effect of UBA1 knockdown on the clonogenic ability of A673 and RDES cells

To investigate the impact of UBA1 on the malignant behaviors of ES cells, we utilized siRNA to target and silence UBA1 expression in A673 and RDES cells. The knockdown efficiency was confirmed by WB analysis (*P* < 0.001, Fig. 4A). Subsequent functional assays, including CCK-8 proliferation and colony formation assays, confirmed that UBA1 knockdown significantly inhibited the proliferation and clonogenic ability of ES cells (*P* < 0.001, Fig. 4B and C).

**Fig. 5:**
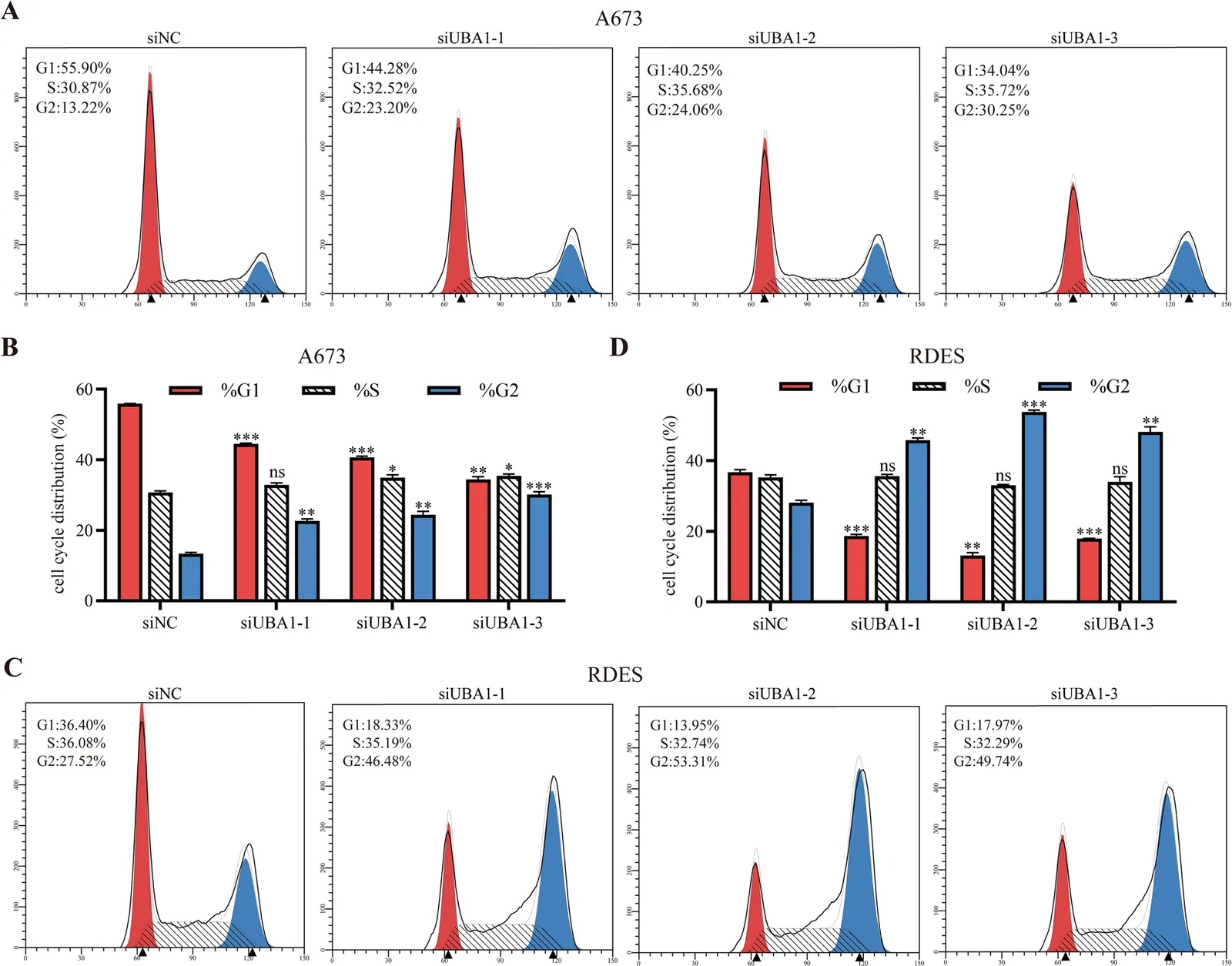
UBA1 knockdown disrupts cell cycle progression in ES cells. Representative flow cytometry histograms (**A**) and quantitative analysis (**B**) of cell cycle distribution in A673 cells following UBA1 knockdown. Representative flow cytometry histograms (**C**) and quantitative analysis (**D**) of cell cycle distribution in RDES cells following UBA1 knockdown

To investigate the mechanism by which UBA1 depletion suppresses proliferation, we examined its impact on cell cycle progression. Flow cytometry analysis revealed that UBA1 knockdown markedly disrupted cell cycle distribution in both A673 and RDES cells, although the extent of this disruption was cell line-specific (Fig. 5A–D). A pronounced G2 phase accumulation (*P* < 0.01), accompanied by a corresponding reduction in the G1 phase, was observed in both cell lines, with the G2 arrest being particularly prominent in RDES cells. In contrast, the S-phase fraction showed a modest increase in A673 cells (from 31% to 33–36%, *P* < 0.05), whereas no significant change was detected in RDES cells.

**Fig. 6:**
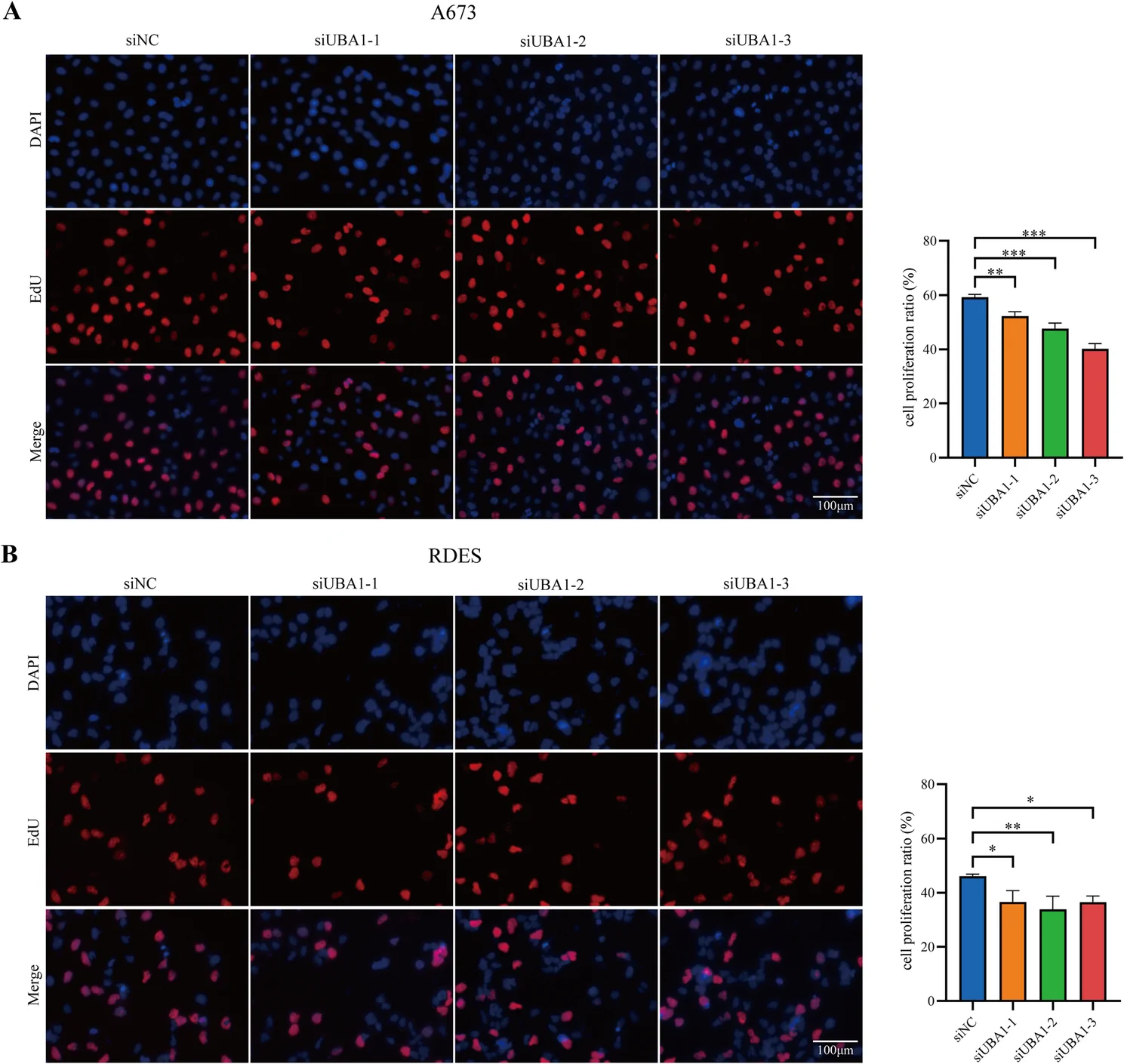
The effect of UBA1 gene knockdown on the proliferation ability of ES cell lines. EdU fluorescence results and statistical analysis graphs of A673 (**A**) and RDES cells (**B**)

Given that G2/M accumulation represents a common and dominant event, we further assessed DNA synthesis activity using EdU incorporation to directly evaluate S-phase progression. Notably, despite the S-phase fraction being either increased (A673) or unchanged (RDES) by flow cytometry, EdU-positive cells were significantly reduced upon UBA1 silencing in both cell lines (*P* < 0.01, Fig. 6A and B). This apparent discrepancy suggests that while the number of cells with S-phase DNA content is not diminished, active DNA replication is compromised, indicative of replication stress and S-phase stalling.

Collectively, these results indicate that UBA1 depletion induces G2/M arrest, consistent with our GSEA findings linking UBA1 to G2/M checkpoint regulation. The concurrent reduction in EdU incorporation further supports that proliferative suppression is driven not only by mitotic arrest but also by impaired DNA replication, particularly under conditions where S-phase cells persist but fail to undergo efficient DNA synthesis.

### UBA1 knockdown attenuates the migratory and invasive capacity of ES cells

**Fig. 7:**
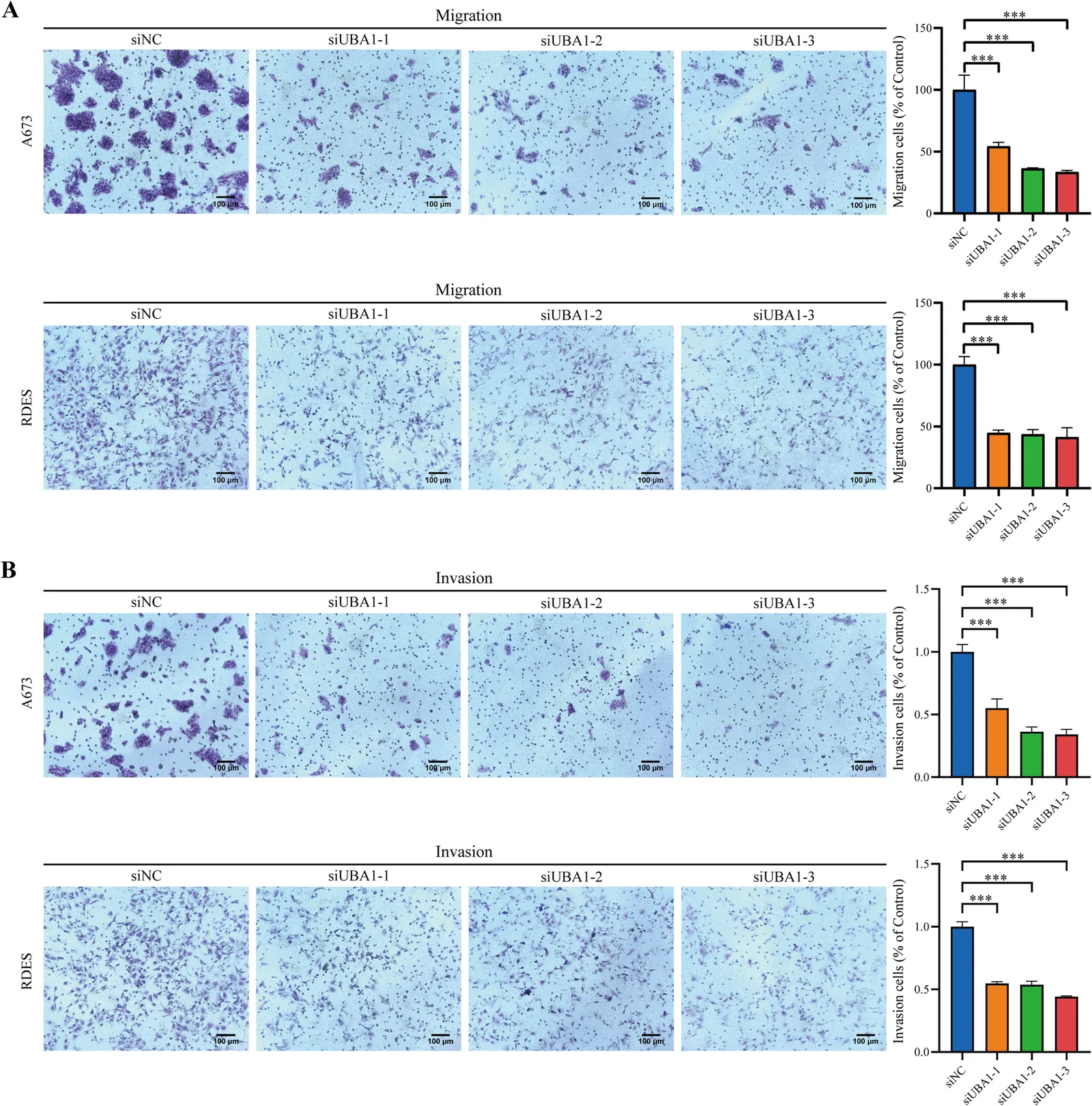
Effect of UBA1 knockdown on the migration and invasion abilities of ES cell lines. **A** Analysis of cell migration by transwell assay. **B** Analysis of cell invasion by transwell assay

We next investigated the impact of UBA1 on the metastatic potential of ES cells. Transwell migration assays revealed that UBA1 knockdown markedly impaired cell motility, significantly reducing the number of migrating cells (*P* < 0.001, Fig. 7A). Furthermore, in Matrigel-based invasion assays, the ability of ES cells to penetrate the extracellular matrix barrier was also substantially attenuated upon UBA1 silencing (*P* < 0.001, Fig. 7B). These results demonstrate that UBA1 is essential for both the migratory and invasive capacities of ES cells, key functional determinants of metastatic dissemination.

### UBA1 knockdown suppresses tumor growth of ES in vivo

**Fig. 8:**
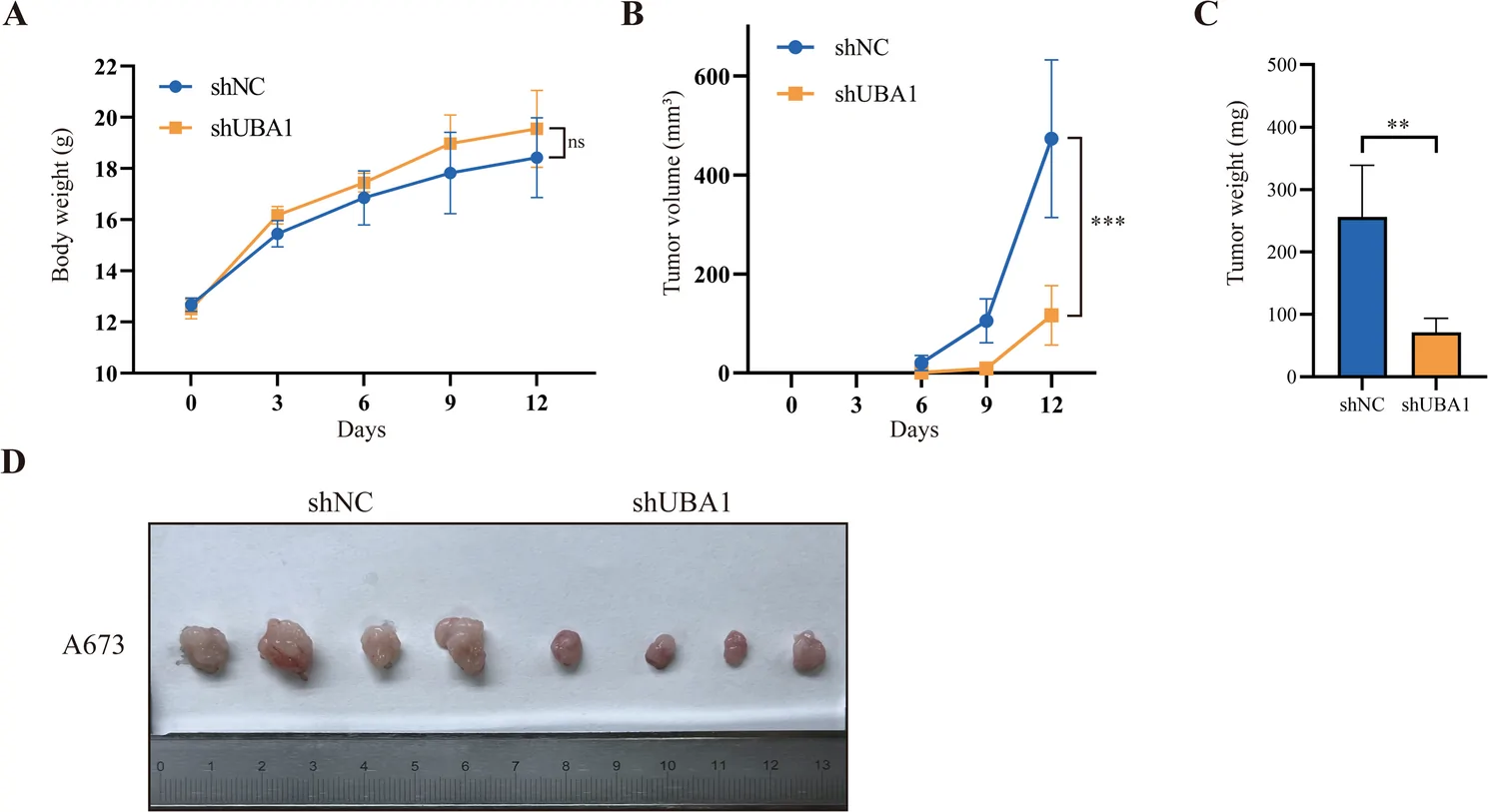
UBA1 knockdown suppresses tumor growth in vivo. **A** Body weight curves of nude mice bearing A673 tumors with stable knockdown of UBA1 (shUBA1) compared to control (shNC). **B** Tumor volume growth curves over the experimental period. **C** Tumor weight at endpoint. **D** Representative photographs of dissected tumors from each group at the end of the experiment

To evaluate the effect of UBA1 on tumor growth in vivo, we first established A673 cell lines with stable UBA1 knockdown and corresponding control cells. Knockdown efficiency was confirmed to meet the requirements of subsequent in vivo experiments. A subcutaneous xenograft model was then established in nude mice to assess tumor growth. UBA1 knockdown significantly inhibited the growth of ES cells, as reflected by marked reductions in both tumor volume and weight compared with the control group (Fig. 8A–D).

### Knockdown of UBA1 leads to G2/M phase arrest

**Fig. 9:**
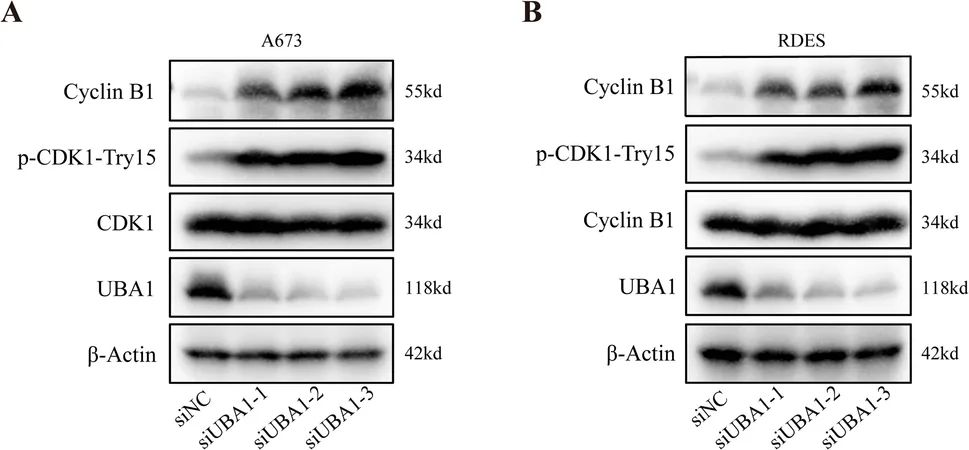
Western blot analysis of G2/M checkpoint proteins after UBA1 knockdown in A673 (**A**) and RDES cells (**B**)

To investigate the molecular mechanism underlying G2/M phase accumulation following UBA1 knockdown in ES cells, we examined the expression levels of key regulators of mitotic entry. Compared with the control group, the siUBA1 group exhibited a significant increase in the inhibitory phosphorylation of CDK1 at Tyr15 (p-CDK1 Tyr15), whereas total CDK1 protein levels remained unchanged. In addition, Cyclin B1 protein expression was markedly upregulated upon UBA1 knockdown. Collectively, these results indicate that UBA1 deficiency is associated with enhanced inhibitory phosphorylation of CDK1 and accumulation of Cyclin B1, consistent with a block in G2/M transition and impaired mitotic progression (Fig. 9A and B).

### TAK-243 recapitulates the anti-tumor effects observed with genetic knockdown

**Fig. 10:**
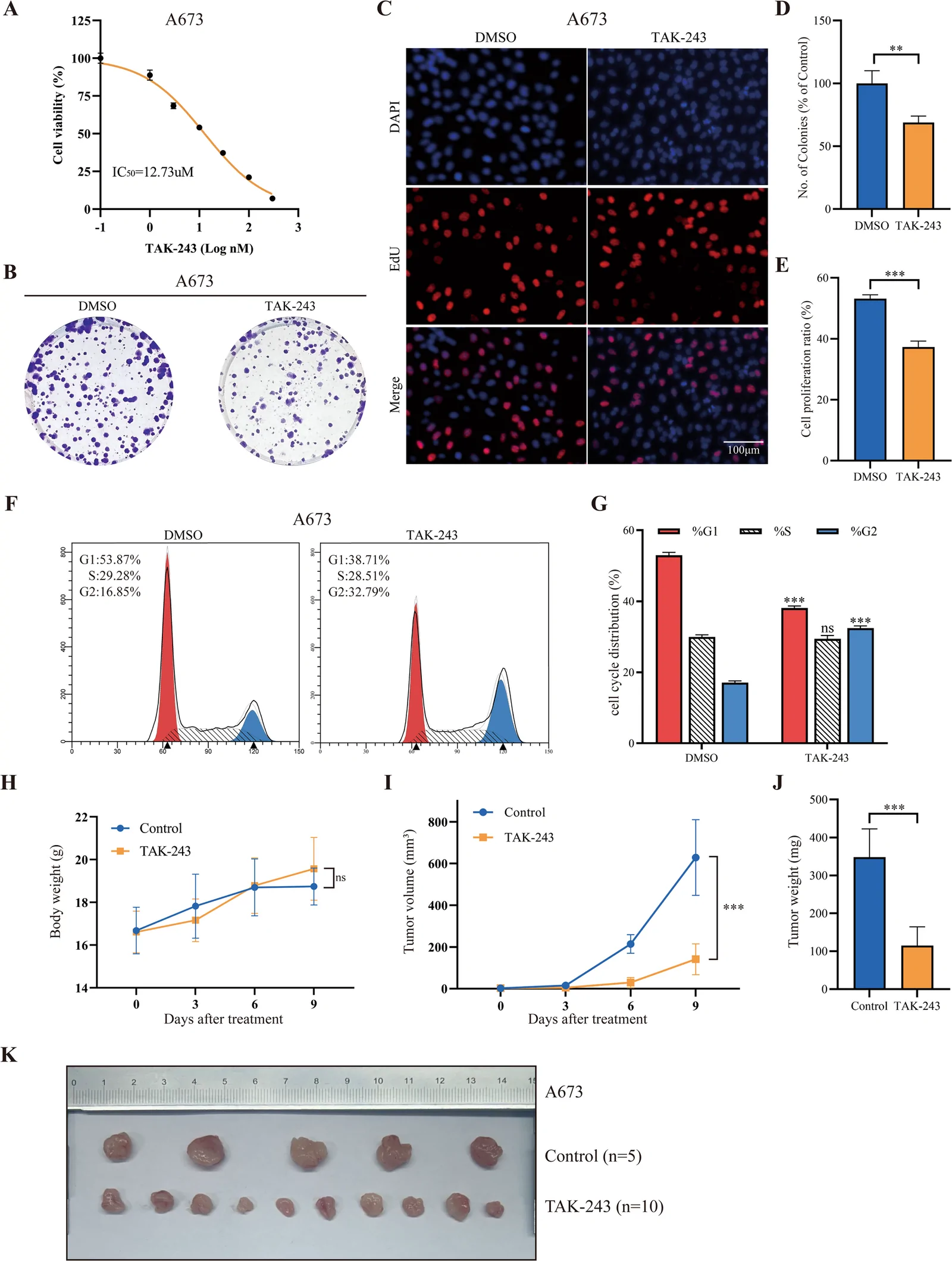
The UBA1 inhibitor TAK-243 suppresses ES cell malignancy in vitro and inhibits tumor growth in vivo. **A** Dose-response curve of A673 cells treated with increasing concentrations of TAK-243 for 24 h. **B** Representative images of colony formation assays in A673 cells treated with DMSO or 20 µM TAK-243. **C** Representative fluorescence microscopy images of EdU incorporation assays under the same treatment conditions. **D** Quantification of colony formation assays. **E** Quantification of EdU-positive cell percentages. **F** Representative flow cytometry histograms of cell cycle distribution in A673 cells treated with DMSO or 20 µM TAK-243. **G** Quantification of cell cycle phase distribution (G0/G1, S, and G2/M) from three independent experiments. **H** Body weight curves of nude mice bearing A673 xenografts treated with vehicle (control) or TAK-243 (10 mg/kg). **I** Tumor volume growth curves measured every three days. **J** Tumor weight at the end of the experiment. **K** Representative photographs of tumors from control and TAK-243-treated mice at the end of the experiment

To validate the therapeutic potential of targeting UBA1 and to determine an appropriate experimental concentration, we first evaluated the sensitivity of A673 cells to the UBA1 pharmacological inhibitor TAK-243. A dose‑response curve generated by CCK‑8 assay after 24 h of treatment revealed an IC_50_ of 12.73 µM (Fig. 10A). Based on this result, a concentration of 20 µM (approximately 1.6‑fold IC_50_) was selected for subsequent in vitro functional assays. Treatment with 20 µM TAK‑243 significantly impaired clonogenic capacity, reducing colony formation to approximately 0.6‑fold of that in DMSO‑treated controls (*P* < 0.01, Fig. 10B and D). EdU incorporation assays further demonstrated that TAK‑243 markedly decreased the proportion of proliferating cells, with the EdU positive rate declining from 55% in the control group to 38% in the treatment group (*P* < 0.01, Fig. 10C and E). Cell cycle analysis by flow cytometry showed that TAK‑243 induced a pronounced G2/M phase accumulation, accompanied by a concomitant reduction in the G1 phase population (*P* < 0.001, Fig. 10F and G). These results closely mirror the phenotypes observed following siRNA‑mediated UBA1 knockdown.

To evaluate the anti‑tumor efficacy of TAK‑243 in vivo, fifteen mice were implanted with tumor cells, and four days later (when tumors became palpable), the mice were randomly assigned to a vehicle control group (*n* = 5) or a TAK‑243 treatment group (*n* = 10). TAK‑243 was administered intraperitoneally at 10 mg/kg every three days. During the treatment period, no significant difference in body weight was observed between the two groups (Fig. 10H). Tumor volumes were measured every three days; starting from day 6 after treatment initiation, tumors in the TAK‑243 group grew significantly more slowly than those in the control group (*P* < 0.001, Fig. 10I). At the end of the experiment, tumor weights were markedly reduced in the TAK‑243‑treated mice compared to controls (*P* < 0.001, Fig. 10J), and representative tumor photographs clearly showed smaller tumors in the treatment group (Fig. 10K). Collectively, these in vivo data corroborate the in vitro findings and demonstrate that pharmacological inhibition of UBA1 effectively suppresses ES tumor growth.

## Discussion

ES represents a formidable clinical challenge, with its aggressive behavior and poorly defined cellular origins necessitating continued efforts to uncover its pathogenic drivers [18]. Central to cellular homeostasis, the UPS has emerged as a critical determinant in cancer biology [19–22]. At the apex of this system sits UBA1, the E1 enzyme that initiates ubiquitination and regulates processes fundamental to tumorigenesis, including cell cycle control, DNA repair, and apoptotic resistance [23–26]. The oncogenic potential of UBA1 is further supported by clinical evidence linking its elevated expression to enhanced proliferation in acute myeloid leukemia and chemoresistance in colorectal cancer [17, 27]. Despite these advances, the involvement of UBA1 in ES development had not been characterized prior to this study.

In the present study, we identified UBA1 as a critical oncoprotein in Ewing sarcoma (ES). UBA1 is significantly overexpressed in ES tissues and correlates with poor patient prognosis. Functional assays demonstrated that UBA1 knockdown suppresses ES cell proliferation, colony formation, migration, and invasion. These findings were further validated by pharmacological inhibition using the UBA1-specific inhibitor TAK‑243, which recapitulated the anti‑proliferative and cell cycle‑arresting effects of genetic knockdown. Moreover, in a subcutaneous xenograft model, UBA1 depletion significantly impaired tumor growth, supporting its oncogenic role in vivo.

Mechanistically, our GSEA predicted enrichment of the G2/M checkpoint pathway in association with high UBA1 expression. Experimental validation revealed that UBA1 knockdown induced a pronounced G2/M phase accumulation, accompanied by increased protein levels of Cyclin B1 and elevated inhibitory phosphorylation of CDK1 at Tyr15—findings that are consistent with impaired G2/M progression. These findings are consistent with studies in Drosophila demonstrating that UBA1 deficiency leads to G2/M arrest due to impaired cyclin B degradation [28, 29]. Furthermore, the reduction in CDC20 protein levels upon UBA1 silencing suggests impaired APC/C activation, which may contribute to the accumulation of Cyclin B1 and failure to exit mitosis [14, 30, 31]. Collectively, these data establish that UBA1 is essential for proper mitotic progression in ES, likely through regulating the stability of key G2/M regulators.

Beyond the G2/M axis, our GSEA also revealed enrichment of the E2F pathway. It is well established that the EWS‑FLI1 fusion protein drives ES pathogenesis by suppressing Rb, leading to E2F activation [32–36]. We speculated that UBA1 might complement this mechanism by facilitating Rb degradation via the ubiquitin‑proteasome system, thereby reinforcing E2F activity [37–39]. Although we did not experimentally validate this axis due to time and resource constraints, this hypothesis remains plausible and warrants future investigation. Similarly, our GSEA unexpectedly showed negative enrichment of the IL‑6/JAK/STAT3 signaling pathway. While sustained STAT3 activation has been reported to promote angiogenesis and chemoresistance in ES [40, 41], our finding raises the possibility that UBA1 may suppress STAT3 activity within immune cells of the tumor microenvironment, potentially contributing to immune evasion [42]. This intriguing hypothesis requires validation in appropriate immune‑competent models.

In summary, our study positions UBA1 as a pleiotropic regulator of cell cycle progression and a promising therapeutic target in ES. The consistent anti‑tumor effects achieved by both genetic knockdown and pharmacological inhibition, together with in vivo evidence, underscore the translational potential of targeting UBA1 in this aggressive malignancy.

## Data Availability

The data generated during the current study are available from the corresponding author upon reasonable request.
